# L'intérêt du dosage de la calcémie devant une tumeur maxillaire: découverte d'une hyperparathyroïdie primitive

**DOI:** 10.11604/pamj.2014.18.315.4967

**Published:** 2014-08-21

**Authors:** Hicham Esselmani, Mounya Bouabdellah, Laila Benchekroun, Sanae Elalami, Najat Handor, Layachi Chabraoui

**Affiliations:** 1 Laboratoire Central de Biochimie Clinique, Hôpital Ibn Sina, Rabat, Maroc; 2 Faculté de Médecine et de Pharmacie de Rabat, Maroc

**Keywords:** Hyperparathyroïdie primitive, hypercalcémie majeure, tumeur brune maxillaire, primary hyperparathyroidism, hypercalcemia, brown maxillary tumor

## Abstract

L'hyperparathyroïdie primaire (HPP) est une affection fréquente, aujourd'hui découverte fortuitement dans 75 à 80% des cas par un dosage systématique de la calcémie biologique. Le diagnostic de cette affection se base sur la mise en évidence concomitante d'une calcémie élevée, d'un taux de parathormone (PTH) élevé (80% des cas) ou normal (20% des cas), et d'une calciurie supérieure à 150 mg/24 heures. Les manifestations squelettiques graves (ostéite fibrokystique, tumeurs brunes, fractures pathologiques) sont rares de nos jours. Les tumeurs brunes constituent un mode de révélation rare de l'hyperparathyroïdie primaire et leur localisation au niveau des maxillaires est exceptionnelle. Nous rapportons ici la longue histoire de la maladie d'un homme de 42 ans atteint d'une hyperparathyroïdie primitive découverte devant une tuméfaction jugale, une hypercalcémie majeure à 3.92 mmol/l et une tumeur brune maxillaire et nous soulignant le rôle du bilan biologique raisonné et de son interprétation en tant que révélateur diagnostique pour orienter le clinicien vers l'hyperparathyroïdie primaire.

## Introduction

L'hyperparathyroïdie constitue actuellement la troisième pathologie endocrinienne après le diabète sucré et la pathologie thyroïdienne [1]. Elle est découverte, dans 75 à 80% des cas, après dosage systématique de la calcémie qui objective l'hypercalcémie qui en découle [2]. Les tumeurs brunes sont des lésions osseuses classiquement retrouvées dans les formes sévères et évoluées d'hyperparathyroïdie primitive [2]. Leur localisation au niveau du massif facial demeure rare et constitue donc un mode exceptionnel de découverte de cette pathologie [3]. Nous rapportons, dans ce travail, un cas original d'hyperparathyroïdie primitive (HPP) révélée par une hypercalcémie majeure associée à une tumeur brune maxillaire. A travers cette observation, nous discutons des aspects physiopathologiques et clinico-biologiques de cette maladie en soulignant le rôle du bilan biologique raisonné et de son interprétation en tant que révélateur diagnostique pour orienter le clinicien vers l'HPP.

## Patient et observation

Il s'agit d'un homme, âgé de 42 ans, hospitalisé en urgence au service d'endocrinologie de l'Hôpital Ibn Sina de Rabat pour une tuméfaction jugale et hypercalcémie. Dans ses antécédents depuis 3 ans, on notait une lithiase rénale bilatérale récidivante. Il y a sept mois, il avait commencé à se plaindre d'une tuméfaction jugale gauche qui a motivé une consultation ORL; l'examen clinique retrouvait alors une tuméfaction au niveau de l'os maxillaire droit, de consistance dure, non douloureuse, mesurant environ 5 cm de grand axe. La radiographie panoramique dentaire et la TDM du massif facial ont révélé de multiples images lytiques maxillaires supérieures et mandibulaires gauches. Cette découverte de a conduit au dosage de la calcémie puis à son hospitalisation en endocrinocrinologie. A son admission, le patient était légèrement asthénique et amaigri; Sur le plan biochimique (Tableau 1), les résultats du laboratoire, ont montré chez notre patient, une calcémie corrigée à 157 mg/l et une phosphorémie à 16 mg/l.

**Tableau 1:** Bilan biochimique sanguin et urinaire du cas présenté

Paramètres	Valeurs retrouvées	Valeurs de référence
**SANG**		
Na (mEq/l)	137	136 - 145
K (mEq/l)	4,4	3.5 – 5.1
Cl (mEq/l)	109	98 – 107
Réserves alcalines (mEq/l)	23	22 – 32
Glycémie (g/l)	0,83	0.7 – 1.10
Urée (g/l)	0,35	0.15 – 0.55
Créatinine (mg/l)	10,4	5.7 – 12.5
Protides (g/l)	65	64 – 83
Albumine (g/l)	38,01	38 - 48
Phosphore (mg/l)	16	30 - 45
Calcium (mg/l)	157	84 - 102
PTH (pg/ml)	1713	10 - 55
Vit D (ng/ml)	14,90	30 - 40
**URINES**		
Calciurie (mg/l)	154	-
Calciurie/24h (mg/24h)	924	100 - 250
Phosphaturie (mg/l)	209	-
Phosphorurie/24h (mg/24h)	1254	300 - 600

Par ailleurs, il n'existait aucune anomalie cardiaque ou neurologique. Les radiographies des genoux, des membres supérieurs et des membres inférieurs n'avaient pas révélés de lésions suspectes écartant d'autres localisations de tumeur brune. Il n'existait pas de syndrome inflammatoire (CRP < 5mg/l). Les paramètres biologiques de routine, à savoir la glycémie, l'urée et la créatinine étaient sans anomalie. Dans les urines, on avait noté une hyperphosphaturie à 1254 mg/24 h et une hypercalciurie à 924 mg/24. Le diagnostic d'hyperparathyroïdie primitive était fortement suspecté. Il a été confirmé par le dosage de la parathormone (1-84) par la technique d'immuno-chimiluminescence microparticulaire (Architect, Abbott^®^) rapportant une augmentation majeure à 1713 pg/dl. L'examen anatomo-pathologique de la biopsie osseuse du maxillaire était en faveur d'une tumeur brune. Les explorations parathyroidiennes (échographie et scintigraphie au MIBI: 1-méthoxy isobutyle isonitrile) ont retrouvé un adénome parathyroïdien polaire inférieur droit volumineux (46 × 25 mm). Par ailleurs, la thyroïde était globalement augmentée de taille avec un aspect hétérogène nodulaire du lobe thyroïdien droit. Une échographie rénale a mis en évidence de multiples microlithiases bilatérales sans dilatation pyélocalicielle.

## Discussion

L'hyperparathyroïdie primitive (HPP) est la conséquence d'une production excessive et inappropriée d'hormone parathyroïdienne ayant pour principale conséquence métabolique une hypercalcémie [4, 5]. Elle est causée par l'existence d'un adénome bénin, unique et sporadique dans 75 à 85% des cas, par une atteinte de plusieurs glandes ou par une hyperplasie dans 15 à 25% des cas. L'association à une néoplasie endocrinienne multiple (NEM I ou II) ou la présence d'un cancer parathyroïdien (< 0,5% des HPP) est très rare [5]. Les patients avec l’ HPP ont en général un calcium total élevé [6] (valeur qui doit être corrigée en fonction de la concentration d'albumine) ou bien un taux élevé du calcium ionisé. Quelques patients souffrant de l'HPP ont des calcémies normales et ne présentent aucun signe d'hyperparathyroïdie secondaire (par ex. secondaire à une carence en vitamine D, une insuffisance rénale, une hypercalciurie nette ou une malabsorption) [7].

Dans l'HPP, le contrôle de la rétroaction de la PTH par le calcium extracellulaire est défectueux. Dans les adénomes parathyroïdiens, les cellules principales perdent leur sensibilité normale au calcium, qui contrôle normalement la sécrétion de PTH par un feed-back négatif [8]. La population cellulaire monoclonale des adénomes parathyroïdiens indique que la pathogenèse résulte d'anomalies dans des gènes contrôlant la prolifération des cellules parathyroïdiennes. La mise en évidence de l'oncogène PRAD1 dans ces adénomes défini par la présence d'un réarrangement chromosomique somatique qui provoque la fusion du promoteur de la PTH avec le gène de la cycline-D1confirme cette hypothèse [8]. L'HPP s'observe chez l'adulte avec un maximum entre 40 à 50 ans (le cas de notre patient). Seulement 2% des cas peuvent se voir avant 30 ans. L'atteinte féminine semble prédominante [5, 9]. Le tableau clinique de l'HPP n'est pas spécifique. La maladie est découverte à l'occasion de signes variables: asthénie, polydipsie, polyurie, constipation, colique néphrétique [5]. Notre malade avait présenté à son admission une asthénie avec un historique de lithiase urinaire bilatérale. L'atteinte rénale avec lithiase n'est actuellement observée que dans 15 à 20% des HPP [10, 11]. Les manifestations osseuses sont rarement révélatrices de la maladie puisqu'elles ne sont présentes qu’à un stade tardif, et se voient dans seulement 2% des cas [2]. Elles associent une résorption osseuse sous-périostée et des tumeurs brunes. Ces dernières se localisent le plus souvent au niveau du squelette axial, l'atteinte mandibulaire est également rapportée contrairement à celle du maxillaire qui est exceptionnelle [3]. Sur le plan histologique, les tumeurs brunes correspondent à une zone d'hyper résorption ostéoclastique contenant un tissu conjonctif inflammatoire hypervasculaire, des cellules géantes, des dépôts d'hémosidérine (d'où le nom de « tumeurs brunes ») et des zones de tissu ostéoïde qui vont remplacer l'os normal [2]. Le principal diagnostic différentiel des tumeurs brunes est constitué par les tumeurs à cellules géantes ou tumeurs à myéloplaxe [3, 5]: il s'agit de tumeurs hypervascularisées, de siège métaphysoépiphysaire, localisées à l'extrémité des os longs, sur le bassin, le sacrum, les vertèbres, dont l'aspect radiologique et surtout histologique peut être parfaitement semblable à celui des tumeurs brunes (comme ce fut le cas chez ce patient): seul le contexte clinico biologique permet alors de faire la distinction entre ces deux types de tumeurs [3].

Cliniquement, les tumeurs brunes peuvent être totalement asymptomatiques, se manifester par des douleurs osseuses ou encore des fractures pathologiques. Au niveau du massif facial, la tuméfaction osseuse représente un mode de révélation classique [3]. Notre patient avait une tuméfaction jugale gauche qui a motivé une consultation ORL ou il a bénéficié d'une panoramique dentaire avec TDM du massif facial objectivant de multiples images lytiques maxillaire supérieure gauches. Cette découverte a conduit au dosage de la calcémie. Le bilan biochimique chez notre patient avait montré une hypercalcémie majeure (calcium corrigé) à 157 mg/l, une hypophosphorémie à 0,60 mmol/l, une augmentation majeure de la parathormone (1713pg/ml) et une baisse de la 25 hydroxy-vitamine D(14 ng/ml). Dans les urines, on retrouvait une hyperphosphaturie à 30 mmol/24 h et une hypercalciurie à 10 mmol/24 h. Le diagnostic de certitude de l'HPP reste essentiellement biologique, orienté par la détermination de plusieurs paramètres biochimiques [12]. Depuis le développement de la mesure automatisée de la calcémie et sa dosage systématique dans la plupart des analyses biologiques, l'HPP est dépistée dans 80% à un stade asymptomatique devant une hypercalcémie ne dépassant pas de plus de 0.25 mmol/l la norme supérieure (donc inférieure à 2.85 mmol/l) [13–15]. La calcémie mesurée sera corrigée par l'albuminémie ou la protidémie à l'aide de la formule: Ca « corrigé » (mmol/L) \= Ca mesuré (mmol/L) + 0,020 ou 0,025 (40 - albumine (g/L)). Cette formule de correction est issue des travaux de Payne publiés en 1973. La calcémie corrigée même modestement augmentée devra être confrontée à une PTH mesurée sur le même échantillon: si la PTH est haute ou normale haute inadaptée à la calcémie, le diagnostic de l'HPP sera suspecté [16]. L'excrétion urinaire de calcium est élevée chez 30 à 40% des patients. L'action rénale de la PTH entraîne en effet une augmentation de la réabsorption tubulaire du calcium [17]. L'hypophosphatémie est classiquement rapportée dans l'HPP, elle est loin d’être constante. La phosphatémie est inférieure à 0,80 mmol/L dans 50 à 70% des cas, rarement supérieure à 1 mmol/L [5]. La prise en compte du couple calcium (en particulier Ca Ionisé)-PTH permet d’évoquer le diagnostic: une Ca élevée associée à une PTH normale-haute est très évocatrice du diagnostic, il s'agit d'une sécrétion inappropriée de PTH [17]. Le dosage de 1,25(OH)^2^ D n'a aucun intérêt dans la démarche diagnostique (un taux supérieur à la norme dans 30% des HPP). L'insuffisance vitaminique D (25OHD) est fréquente par l'accélération de la conversion de la 25OHD en 1,25(OH)^2^ D et en composés 24 hydroxylés [18]. Les explorations parathyroïdiennes (échographie, scintigraphie au MIBI) ont retrouvé un volumineux adénome parathyroïdien polaire inférieur droit mesurant 46 × 25 mm. L’échographe rénale a mis en évidence de multiples micro lithiase bilatérales.

Devant toutes ces données cliniques, biologiques et radiologiques le diagnostic retenu a donc été celui d'hyperparathyroïdie primitive compliquée de lithiase urinaire bilatérale avec tumeur brune maxillaire. Le traitement de la tumeur brune est dépendant de l’évolution des paramètres biochimiques après extirpation de la tumeur parathyroïdienne. Habituellement, les tumeurs brunes régressent spontanément, complètement ou partiellement [19]. Aucun traitement médical de l'HPP n'est satisfaisant. Le traitement médical est réservé à des HPP symptomatiques qui ne peuvent être opérées et peut se discuter dans des HPP asymptomatiques [5]. Le traitement de choix de l'HPP est aujourd'hui la chirurgie parathyroïdienne [5, 20]. Devant l'hypercalcémie majeure, notre malade a bénéficié d'une réhydratation par le sérum salé associées à l'administration du furosémide et il est programmé pour l'exérèse chirurgicale de l'adénome.

## Conclusion

L'observation d'une tumeur maxillaire conduit à évoquer différentes pistes étiologiques telles que les tumeurs brune et les tumeurs à cellules géantes, un bilan biochimique a minima qui repose sur l'association d'une hypercalcémie, d'un taux de parathormone normal ou élevé, et d'une calciurie supérieure à 150 mg/24 heures permettra au clinicien de faire la distinction et de poser diagnostic de certitude de l'hyperparathyroïdie primitive compliquée d'une tumeur brune maxillaire. À la lumière de cette observation sur l'hyperparathyroïdie nous soulignons aussi: le retard diagnostique en dépit des nombreux signes cliniques (asthénie, coliques néphrétiques), la révélation exceptionnelle de l'hyperparathyroïdie primitive par les tumeurs brunes et la rareté de la localisation maxillaire des tumeurs brunes.
